# HIF1α mediates circadian regulation of skeletal muscle metabolism and substrate preference in response to time-of-day exercise

**DOI:** 10.1073/pnas.2504080122

**Published:** 2025-07-08

**Authors:** Amy M. Ehrlich, Kirstin A. MacGregor, Stephen P. Ashcroft, Lewin Small, Ali Altıntaş, Alexander V. Chibalin, Matthias Anagho-Mattanovich, Ben Stocks, Thomas Moritz, Jonas T. Treebak, Juleen R. Zierath

**Affiliations:** ^a^Novo Nordisk Foundation Center for Basic Metabolic Research, Faculty of Health and Medical Sciences, University of Copenhagen, Copenhagen DK-2200, Denmark; ^b^Integrative Physiology Department of Molecular Medicine and Surgery, Karolinska Institutet, Stockholm SE-171 77, Sweden; ^c^Integrative Physiology Department of Physiology and Pharmacology, Karolinska Institutet, Stockholm SE-171 77, Sweden

**Keywords:** metabolism, circadian, energy metabolism, exercise, transcription factor

## Abstract

The regulation of metabolism in peripheral tissues is intricately linked to circadian rhythms, with hypoxia-inducible factor-1α (HIF1α) implicated in modulating time-of-day-specific exercise responses. To investigate this relationship, we generated a skeletal muscle-specific HIF1α knockout (KO) mouse model and performed extensive metabolic phenotyping and transcriptomic profiling under both basal conditions and following acute exercise during early rest (ZT3) and active (ZT15) phases. Our findings reveal that HIF1α drives a more robust transcriptional and glycolytic response to exercise at ZT3, promoting glucose oxidation and mannose-6-phosphate production while potentially sparing fatty acid oxidation. In the absence of HIF1α, skeletal muscle metabolism shifts toward oxidative pathways at ZT3, with notable alterations in glucose fate. These results establish HIF1α as an important regulator of time-of-day-specific metabolic adaptations, integrating circadian and energetic signals to optimize substrate utilization. This work highlights the broader significance of HIF1α in coordinating circadian influences on metabolic health and exercise performance.

## Significance

The timing of exercise influences metabolic outcomes, yet the molecular mechanisms underlying time-of-day-specific adaptations remain unclear. Our study identifies hypoxia-inducible factor-1α (HIF1α) as a key regulator of circadian-dependent metabolic responses in skeletal muscle. Using a muscle-specific HIF1α knockout mouse model, we demonstrate that HIF1α enhances glycolytic metabolism during early rest-phase exercise, optimizing glucose utilization while suppressing fatty acid oxidation. Loss of HIF1α shifts metabolism toward oxidative pathways, disrupting this phase-specific adaptation. These findings reveal a critical role for HIF1α in integrating circadian and metabolic signals, with implications for optimizing exercise timing to improve metabolic health and performance.

Physical activity is a cornerstone of health, reducing the risk of all-cause mortality and serving as an important strategy for the prevention and management of chronic diseases (1, 2). Exercise confers systemic benefits, but its effects are particularly pronounced within skeletal muscle, where it promotes insulin-independent glucose uptake, enhances insulin sensitivity, and drives transcriptional programs that support metabolic homeostasis (3–5). These effects underscore the role of skeletal muscle as a central organ in mediating the metabolic benefits of exercise. While physiological factors, including exercise intensity, duration, and nutritional status, influence these molecular adaptations, emerging research highlights the timing of exercise as a critical determinant of both systemic and skeletal muscle-specific metabolic responses (6–10). Evidence suggests that exercise performed at different times of day yields distinct metabolic and transcriptional outcomes (11, 12). However, the molecular mechanisms driving these circadian-dependent variations in exercise adaptations remain largely unexplored.

The circadian clock is a cell-autonomous transcriptional feedback loop that regulates rhythmicity in biological processes across the 24-h day-night cycle. At the molecular level, the core clock transcription factors BMAL1 and CLOCK, activate *Period* and *Cryptochrome* genes, which in turn inhibit their own transcription through a tightly regulated feedback mechanism (13). This circadian program operates at both central and peripheral levels; the suprachiasmatic nucleus acts as a master regulator, synchronizing peripheral clocks in metabolically active tissues via humoral and neural cues. While light serves as the dominant zeitgebers, energetic stressors, such as food intake and physical activity, also entrain peripheral clocks, independently of central mechanisms (14, 15).

In skeletal muscle, the circadian clock orchestrates daily fluctuations in glucose and lipid metabolism, insulin sensitivity, and thermogenesis, all of which influence exercise performance (12, 16, 17). Diurnal variations in exercise capacity are evident in both rodents (9) and humans (18), with striking time-of-day-dependent differences in the transcriptional and metabolic response to acute exercise (7, 8). In rodents, exercise performed during the early active phase promotes a catabolic state characterized by increased reliance on fatty acids, amino acids, and ketones (7, 19). This phase-specific response is accompanied by an accumulation of hypoxia-inducible factor-1 α (HIF1α) protein and the activation of its downstream targets in skeletal muscle (7). Notably, HIF1α serves as both a regulator of glycolytic and oxidative metabolism and a modulator of circadian rhythms via a direct interaction with core clock components (20).

Despite these insights, the role of HIF1α in mediating time-of-day-dependent exercise responses has not been investigated. To address this, we generated a skeletal muscle-specific *HIF1α* knockout mouse model (*Hsa-cre;Hif1α*^fl/fl^, HIF1α KO), by utilizing human α-skeletal actin (HSA) Cre recombinase to mediate excision of floxed *HIF1α* alleles (*HIF1α*^fl/fl^, fl/fl). We then characterized the metabolic and transcriptional responses of these mice to acute exercise across the diurnal cycle. We hypothesized that skeletal muscle HIF1α is necessary for the distinct metabolic adaptations observed during exercise in the early active phase, when HIF1α expression peaks in wild-type (WT) mice. Contrary to this hypothesis, our findings reveal that the loss of HIF1α disrupts the glycolytic response to exercise in the early rest phase, resulting in a systemic metabolic shift toward fatty acid oxidation while leaving the core components of the skeletal muscle clock unaffected. These results underscore the role of HIF1α in mediating the interplay between metabolic states and circadian rhythms during exercise.

## Results

### HIF1α KO Mice Maintain Normal Behavioral Rhythmicity.

**Fig. 1.**
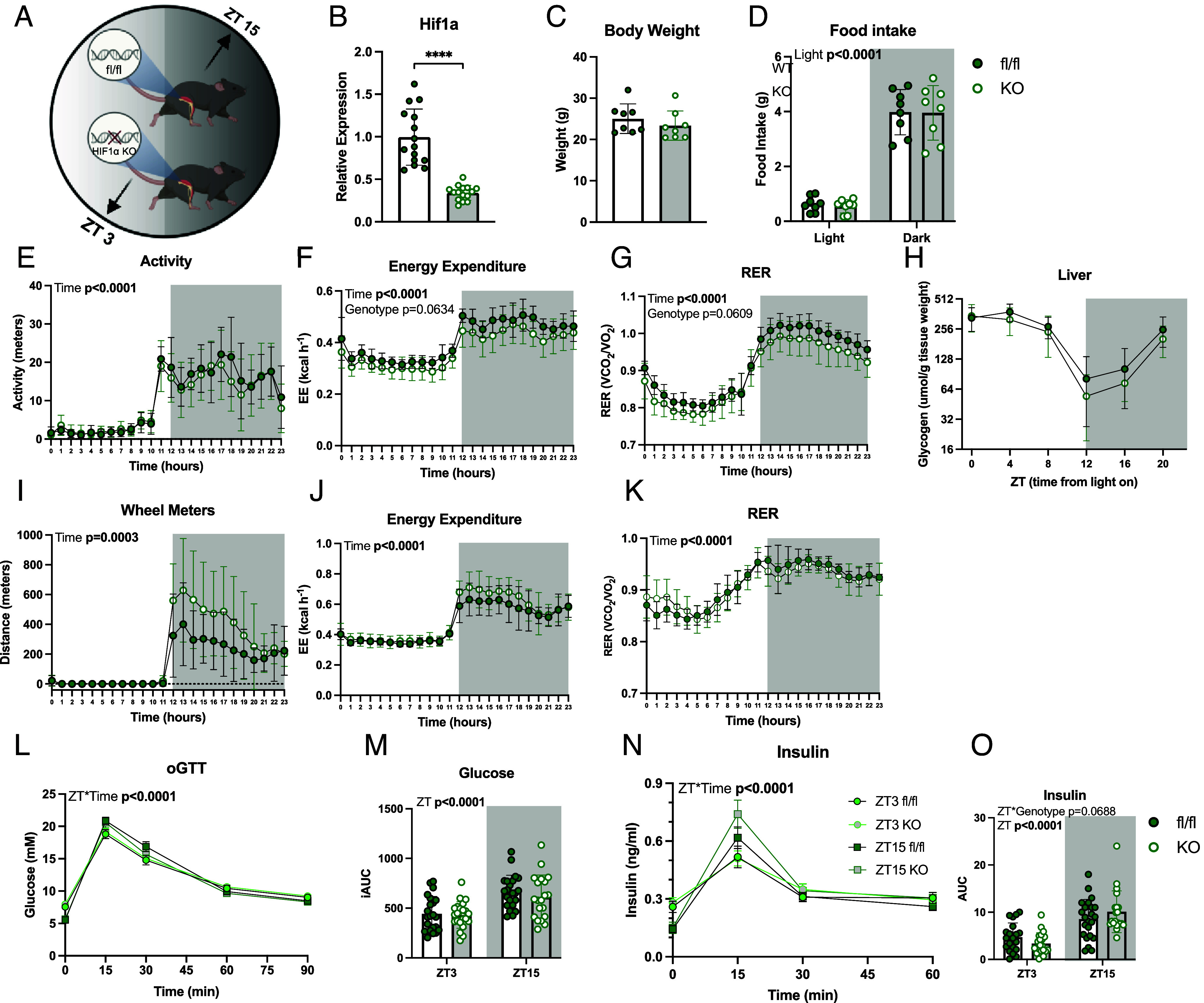
Metabolic phenotyping of Hif1α KO mice. (*A*) Schematic of the skeletal muscle HIF1α KO model. (*B*) *Hif1α* gene expression in quadriceps muscle bulk tissue from fl/fl and HIF1α KO mice (n = 14-16; males). (*C*) Body weight; (n = 8). (*D*) Average food intake during the light phase (ZT0-12) and dark phase (ZT12-24); (n = 8). (*E*) Activity trace over the course of a full 24-h period; (n = 8). Data show average values over hourly intervals. (*F*) EE trace across the 24-h period; (n = 8). Data show average values over hourly intervals. (*G*) RER trace across the 24-h period; (n = 8). Data show average values over hourly intervals. (*H*) Liver glycogen was measured every 4 h over a 24-h period. (*I*) Average distance run on voluntary running wheels across the 24-h light–dark cycle and cumulative meters run during the light and dark phases; (n = 4-8). (*J*) EE across the 24-h light–dark cycle in mice with access to running wheels; (n = 4-8). (*K*) RER across the 24-h light–dark cycle in mice with access to running wheels; (n = 4-8). Values from (*I*–*K*) are an average across a 5-d period. (*L*) Blood glucose measurements collected across a 90 min oGTT conducted at either ZT3 or ZT15; (n = 22-24). (*M*) iAUC of glucose from the oGTT; (n = 22-24). (*N*) Blood insulin measurements occurring during an oGTT at ZT3 and ZT15; (n = 22-24). (*O*) AUC of insulin from oGTT; (n = 22-24). ZT refers to mice undergoing the experiment at ZT3 versus ZT15. Time refers to the subsequent samples obtained from mice undergoing experiment at either ZT3 or ZT15. Genotype refers to fl/fl versus KO mice. Light refers to the summation or average of samples occurring in light phase versus the dark phase. Data are presented as mean values ± the SEM. Statistical significance was tested by unpaired two-tailed Student’s *t* test or two-way ANOVA with the Holm–Šídák post hoc test; *P < 0.05, **P < 0.01, ***P < 0.001, ****P < 0.0001. Significant *P*-values in bold text.

To investigate the role of HIF1α in the circadian regulation of metabolism and the time-of-day-dependent exercise response, we generated skeletal muscle-specific HIF1α knockout mice (*Hsa-cre;Hif1α*^fl/fl^, HIF1α KO) (Fig. 1*A*). After confirming the reduction in skeletal muscle *Hif1α* transcripts in HIF1α KO mice compared to *Hif1α*^fl/fl^ (fl/fl) littermates (Fig. 1*B*), we next assessed systemic metabolic alterations and potential disruptions to circadian rhythms. HIF1α KO mice displayed no differences in body weight, muscle mass, food intake, or activity compared to fl/fl mice during both the light (inactive) phase and dark (active) phases (Fig. 1 *C*–*E* and *SI Appendix*, Fig. S1 *A–D*). As anticipated, energy expenditure (EE), food intake, and activity were all elevated during the dark phase in both genotypes (Fig. 1 *D*–*F* and *SI Appendix*, Fig. S1 *D* and E). Though HIF1α KO mice exhibited nonsignificant reductions in the average hourly EE across the 24-h period, there was a significant effect of genotype on the cumulative EE in the light and dark phases (Fig. 1*F* and *SI Appendix*, Fig. S1*E*). Similarly, the hourly average respiratory exchange ratio (RER) was nonsignificantly reduced across the 24-h period in HIF1α KO mouse concurrent with a significant effect of genotype on the cumulative carbon dioxide production and oxygen consumption (Fig. 1*G* and *SI Appendix*, Fig. S1 *F*–J). Hepatic and skeletal muscle glycogen levels, as well as plasma levels of nonesterified fatty acids (NEFA) and triglycerides (TG), were comparable between genotypes throughout the circadian cycle (Fig. 1*H* and *SI Appendix*, Fig. S1 *K*–M).

To further examine circadian behavior, we provided mice with voluntary access to running wheels. Over a 5-d recording period following acclimatization, running distance and diurnal patterns were indistinguishable between genotypes, with most activity confined to the dark phase (Fig. 1*I* and *SI Appendix*, Fig. S1*N*). Additionally, when access to a running wheel was allowed, there were no differences in either EE or RER between genotypes (Fig. 1 *J* and *K* and *SI Appendix*, Fig. S1 *O* and P). These findings provide evidence that skeletal muscle-specific HIF1α ablation does not disrupt behavioral rhythmicity or circadian regulation of energy metabolism when measured under standard conditions of 12 h in the light phase and 12 h in the dark phase.

### Glucose Tolerance Varies by Time of Day, Independent of HIF1α Expression.

To assess glucose handling and its relationship to HIF1α, we conducted oral glucose tolerance tests (oGTTs) during two distinct metabolic states. The first state, the early rest phase (ZT3), is characterized by preferential carbohydrate oxidation due to replete liver glycogen stores. The second state, the early active phase (ZT15), is marked by a metabolic shift toward increased lipid oxidation, reflecting depleted liver glycogen levels. Blood glucose and insulin levels were elevated to a greater extent at ZT15 compared to ZT3, irrespective of genotype (Fig. 1 *L*–*O*). The reduced glucose incremental area under the curve (iAUC) at ZT3 reflected a combination of lower basal blood glucose and higher peak blood glucose at ZT15 (Fig. 1 *L* and *M*). These findings highlight time-of-day-specific variations in glucose tolerance that occur independently of HIF1α.

### Skeletal Muscle Molecular Rhythmicity Remains Intact in HIF1α KO Mice.

Given the integral role of HIF1α in circadian regulation through interactions with core clock factors such as *Bmal1, Per2,* and *Cry2* (21–23), we hypothesized that skeletal muscle clock gene expression would be disrupted in HIF1α KO mice in the basal state. However, rhythmic expression of key clock genes (*Bmal1, Clock, Nr1d1, Dbp, Per1,* and *Per2)* was preserved in HIF1α KO mice, with *Cry1* rhythmic only in fl/fl mice. Differential rhythmicity testing revealed no significant differences in molecular rhythmicity between genotypes (*SI Appendix*, Fig. S2). Thus, the absence of skeletal muscle HIF1α does not affect the messenger RNA (mRNA) expression of genes within the core clock machinery.

### HIF1α Ablation Reduces Lactate Production during Graded Exercise.

**Fig. 2.**
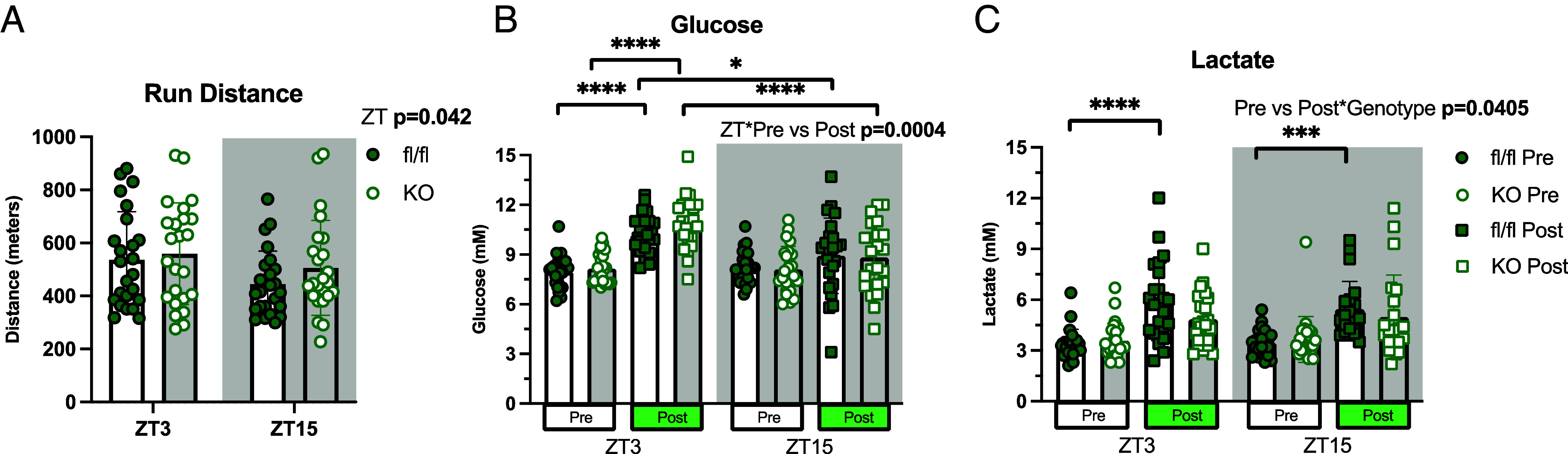
Time-of-day graded exercise test assessment. (*A*) Average running distance during a graded exercise test at ZT3 and ZT15; (n = 24). (*B*) Blood glucose measurements immediately before (pre) and after (post) an exercise test performed at ZT3 and ZT15; (n = 24). (*C*) Lactate measurements prior to pre- and post-graded exercise test; (n = 24). Pre versus Post refers to samples collected immediately prior versus immediately after the exercise bout. Genotype refers to fl/fl versus KO, ZT refers to ZT3 versus ZT15. Data are presented as mean values ± the SEM. Statistical significance was tested by two-way or three-way ANOVA with the Holm–Šídák post hoc test; *P < 0.05, **P < 0.01, ***P < 0.001, ****P < 0.0001.

To investigate exercise performance and metabolism, mice underwent graded exercise testing at ZT3 and ZT15. Both fl/fl and HIF1α KO covered more distance and achieved faster running speeds at ZT3 than ZT15 (Fig. 2*A*), indicating improved performance in the early rest phase. Exercise elevated blood glucose levels at ZT3, but not at ZT15, with similar responses observed in both genotypes (Fig. 2*B* and *SI Appendix*, Fig. S3). As anticipated, lactate levels increased robustly in fl/fl mice following exercise (final) at either timepoint, compared to the pre-exercise (initial) levels. However, this elevation in lactate was not observed in HIF1α KO mice at either timepoint, suggesting a diminished reliance on glycolytic metabolism compared to fl/fl mice (Fig. 2*C* and *SI Appendix*, Fig. S3).

### Time-of-Day and HIF1α Modulate Glucose Homeostasis Postexercise.

**Fig. 3.**
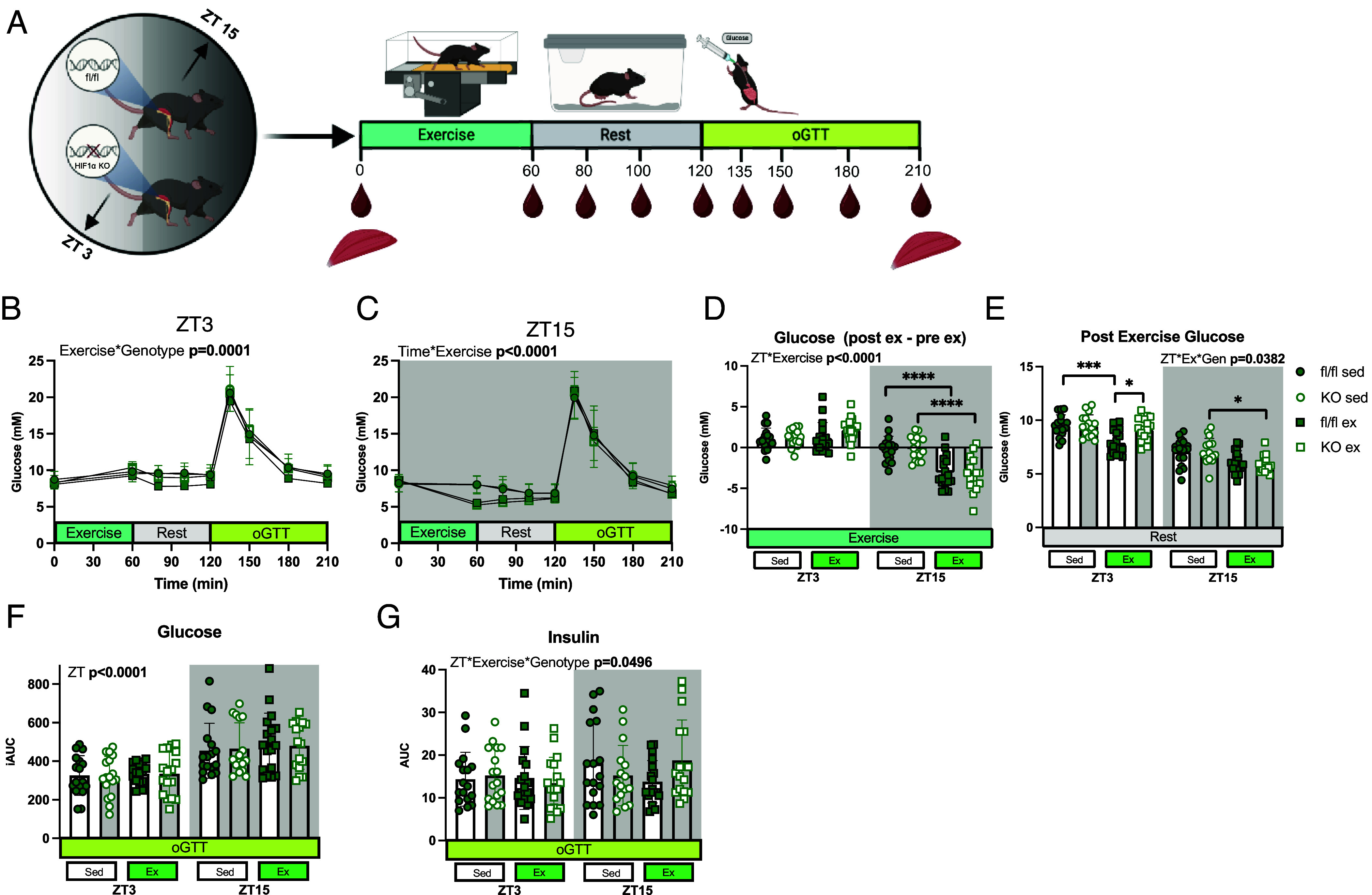
Metabolic response to an acute bout of exercise at ZT3 versus ZT15. (*A*) Schematic of experimental design. (*B*) Blood glucose measurements at ZT3 and (*C*). Blood glucose measurements at ZT15 before and after an acute exercise bout, during the rest period following the exercise, and during a 90 min oGTT; (n = 16-18). (*D*) Change in blood glucose from exercise (pre-exercise blood glucose; timepoint 0 min, subtracted from post exercise blood glucose; timepoint 60 min) at ZT3 and at ZT15. (*E*) Average blood glucose measurements during the rest period following exercise. (*F*) Blood glucose AUC from oGTT at ZT3 and ZT15. (*G*) Insulin AUC from oGTT. ZT refers to mice undergoing the experiment at ZT3 versus ZT15. Time refers to the subsequent samples obtained from mice undergoing experiment at either ZT3 or ZT15. Genotype (Gen) refers to fl/fl versus KO mice. Exercise (Ex) refers to exercised mice versus sedentary control mice. Data are presented as mean values ± the SEM. Statistical significance was tested by two-way or three-way ANOVA with the Holm–Šídák post hoc test; *P < 0.05, **P < 0.01, ***P < 0.001, ****P < 0.0001.

To assess the combined effects of time-of-day and exercise on glucose handling, we measured glucose dynamics following a 1-h treadmill run preceding an oGTT (Fig. 3*A*). Blood glucose reductions occurred immediately postexercise at ZT15 for both genotypes, whereas at ZT3, all mice had an initial increase in blood glucose levels (Fig. 3 *B* and *C*). At ZT3, blood glucose levels dropped only in exercised fl/fl mice, while they remained elevated in HIF1α KO mice (Fig. 3 *D* and *E*). This observation suggests that exercise induces altered glucose metabolism in the absence of HIF1α.

Glucose dynamics resulting from the oGTT differed by time of day, but not by genotype (Fig. 3*F*). However, a three-way interaction was observed between time of day (ZT), exercise, and genotype in the insulin response (Fig. 3*G* and *SI Appendix*, Fig. S4). At the 15-min timepoint, a two-way interaction between exercise and genotype was detected, with a trend toward elevated insulin levels in exercised HIF1α KO mice compared to exercised fl/fl mice; however, post hoc comparisons did not reach statistical significance. These results suggest a potential difference in insulin dynamics at ZT15, with exercised HIF1α KO mice requiring higher insulin levels to achieve the same blood glucose concentration as sedentary HIF1α KO mice and fl/fl mice (Fig. 3*G* and *SI Appendix*, Fig. S4). Glycogen metabolism in skeletal muscle and liver, as well as plasma NEFA levels, were comparable between genotypes in response to exercise though HIF1α KO mice, at ZT15, replenished glycogen to a greater degree than fl/fl mice in the gastrocnemius (*SI Appendix*, Fig. S5 *A*–D).

**Fig. 4.**
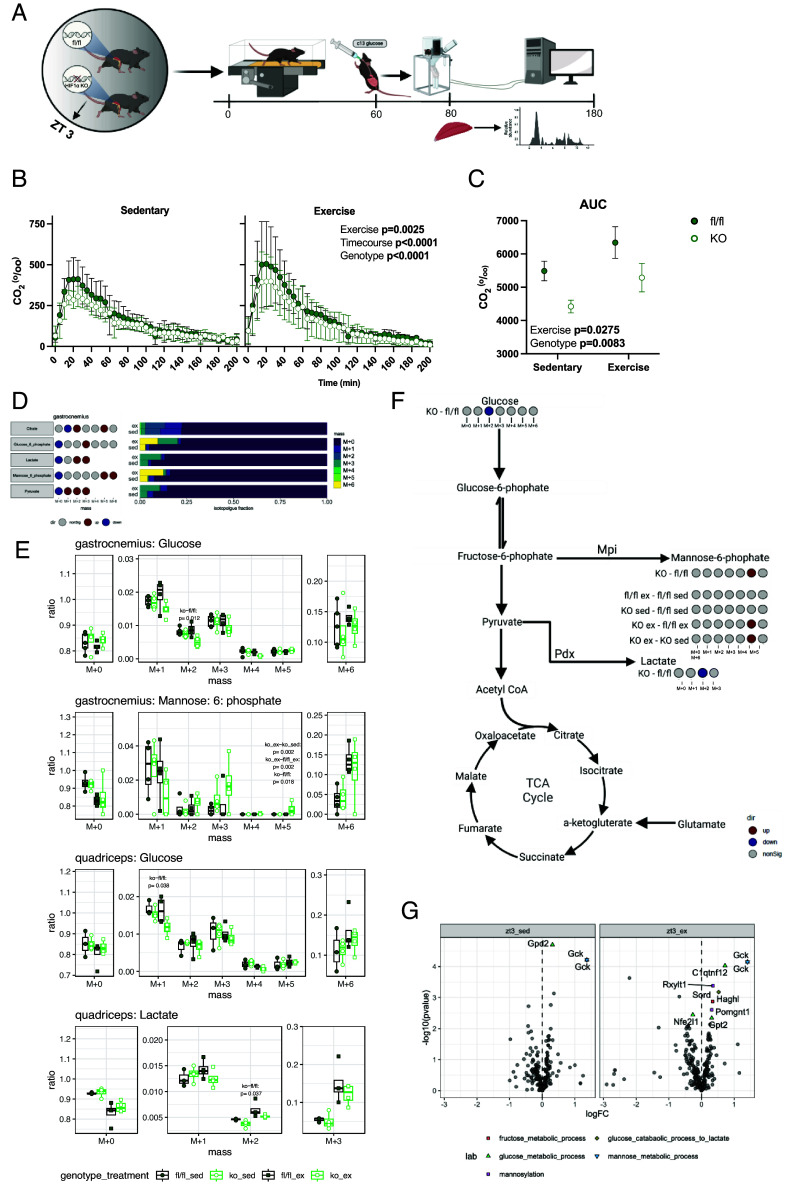
Metabolic fate of a glucose load following an exercise at ZT3. (*A*) Overview of the experimental procedures. (*B*) Whole body glucose oxidation measured via exhaled ^13^CO_2_ and (*C*). Accumulative whole body glucose oxidation following a 1 h acute exercise bout. (*D*) Effect of acute exercise on ^13^C fractions of metabolites gastrocnemius muscle following ^13^C-glucose administration (n = 8). Gray circles indicate no significant changes in isotopologue between sedentary (fl/fl and KO mice) and exercised (fl/fl and KO mice) and red circles indicate a significant increase while blue indicate a significant decrease. (*E*) Altered ^13^C fractions of metabolites in skeletal muscle between fl/fl and HIF1α KO mice in sedentary and exercised conditions. (*F*) Summary of significantly altered ^13^C fractions of metabolites between fl/fl and HIF1α KO mice in sedentary and exercised conditions in skeletal muscles (n = 8). (*G*) mRNA expression levels of genes in the glycolytic pathway differentially expressed in fl/fl and HIF1α KO mice at ZT3 in sedentary and exercised conditions (n = 8). Data are presented as mean values ± the SD. Statistical significance was tested by two-way or three-way ANOVA with the Holm–Šídák post hoc test; *P < 0.05, **P < 0.01, ***P < 0.001, ****P < 0.0001.

To investigate the observed differences in blood glucose following treadmill exercise at ZT3, we selected this timepoint to measure glucose oxidation postexercise. Immediately after running, mice received an oral bolus of U^13^C-glucose, and expired ^13^CO_2_ was measured (Fig. 4*A*). Exercise increased glucose oxidation in both genotypes; however, HIF1α KO mice exhibited reduced glucose oxidation both with exercise and at rest (Fig. 4 *B* and *C*). To further elucidate the mechanism underlying the reduced glucose oxidation in HIF1α KO mice at ZT3, tissues were collected 20 min postexercise following intravenous administration of U^13^C-glucose. The isotopologues are presented to show which specific carbon positions were isotopically labeled in the measured metabolites, allowing us to trace the metabolic fate of the administered glucose. As expected, exercise stimulated glycolytic metabolism in both fl/fl and KO mice, reflected by a reduction in the fraction of unlabeled (m + 0) isotopologues and an increase in labeled glucose-6-phosphate (m + 3), lactate (m + 2, m + 3), and pyruvate (m + 1, m + 2, m + 3) in the quadriceps muscle (Fig. 4*D*). In HIF1α KO mice, however, the fraction of labeled glucose (m + 1, m + 2, and m + 5) and lactate (m + 2) was reduced compared to fl/fl mice (Fig. 4 *E* and *F* and *SI Appendix*, Fig. S6). Conversely, the fraction of labeled mannose-6-phosphate (m + 5) was increased in HIF1α KO mice under both sedentary and exercise conditions, as well as compared to fl/fl mice during exercise (Fig. 4 *E* and *F*). These findings suggest that glucose utilization is diminished in HIF1α KO mice, accompanied by a shift toward increased mannose-6-phosphate metabolism during exercise in the early rest phase. Supporting this, mRNA expression of genes involved in fructose, glucose, and mannose metabolic pathways were elevated in HIF1α KO compared to fl/fl mice, including *Mpi*, which encodes the isomerase responsible for converting fructose-6-phosphate to mannose-6-phosphate (Fig. 4 *F* and *G* and *SI Appendix*, Fig. S6*B*).

### HIF1α KO Induces a Shift toward Oxidative Metabolism in Skeletal Muscle.

**Fig. 5.**
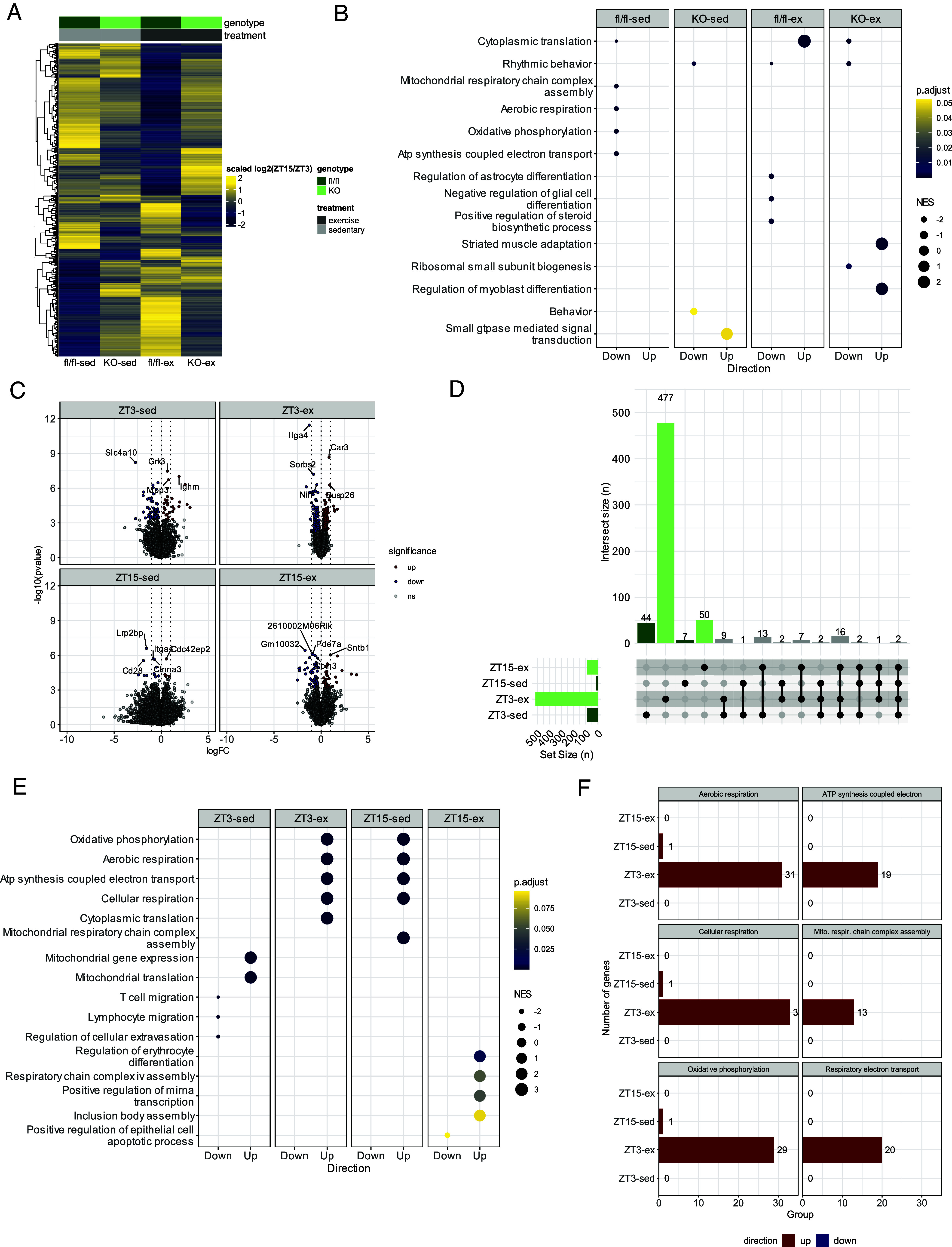
Transcriptomic analysis of gastrocnemius muscle following exercise at ZT3 and ZT15. (*A*) Heatmap showing differentially expressed transcripts between ZT3 and ZT15. (*B*) Gene ontology analysis showing the top 7 biological processes up or downregulated between ZT 3 and ZT 15. Gene set enrichment analysis was performed on genes ranked by FDR. (*C*) Differential expression of logFC between fl/fl and HIF1α KO mice. (*D*) Number of differentially expressed genes between fl/fl and HIF1α KO mice and the intersection of these transcripts. (*E*) Gene ontology analysis showing the top 7 biological processes up or downregulated between fl/fl and HIF1α KO mice. Gene set enrichment analysis was performed on genes ranked by FDR. (*F*) Number of differentially expressed genes within the top 6 biological processes related to aerobic metabolism and mitochondrial function.

To gain insight into transcriptional adaptations underlying the observed metabolic phenotypes, we conducted transcriptomic analysis of the gastrocnemius muscle collected after a 60 min acute exercise bout occurring at either ZT3 or ZT15 from sedentary and exercised mice. HIF1α KO mice exhibited distinct transcriptional profiles, with 724 transcripts upregulated and 749 downregulated compared to fl/fl mice (*SI Appendix*, Fig. S7*A*). Gene ontology analysis revealed an enrichment of pathways related to oxidative metabolism and mitochondrial function in HIF1α KO mice, aligning with the increased reliance on oxidative metabolism (*SI Appendix*, Fig. S7*B*). Notably, transcriptional differences between ZT3 and ZT15 in both exercise and sedentary states were quantitatively greater in fl/fl mice (sedentary = 451, postexercise = 273) compared to HIF1α KO mice (sedentary = 101, exercise = 191) (Fig. 5*A*). Among these, 317 transcripts were unique to sedentary fl/fl mice and 193 were unique to postexercise fl/fl mice, suggesting that HIF1α is essential for time-of-day-dependent transcriptional regulation. Supporting this, gene ontology analysis identified enrichment of pathways associated with oxidative metabolism and mitochondrial function between ZT15 and ZT3, but exclusively in sedentary fl/fl mice (Fig. 5*B*). These findings provide further evidence for the role of HIF1α in mediating a diurnal metabolic switch in sedentary conditions.

In exercised mice, a quantitatively greater number of transcripts were differentially expressed between HIF1α KO and fl/fl mice at ZT3 (N = 516) compared to ZT15 (N = 91) (Fig. 5 *C* and *D*). Within these differentially expressed transcripts, 477 were unique to exercise at ZT3 and 50 were unique to exercise at ZT15 (Fig. 5*D*). Conversely, in sedentary mice, similar skeletal muscle transcriptional profiles were observed between HIF1α KO and fl/fl mice at ZT3 and ZT15 (Fig. 5*D*). Gene set enrichment analysis of transcriptional profiles between HIF1α KO and fl/fl mice in response to exercise identified a positive enrichment of gene ontology terms involved in oxidative metabolism (oxidative phosphorylation, aerobic respiration, cellular respiration) and mitochondrial function (mitochondrial respiratory chain complex assembly, ATP synthesis coupled electron transport and respiratory electron transport chain) at ZT3, but not ZT15 (Fig. 5 *E* and *F*). These data indicate the presence of an interaction between HIF1α and time-of-day on the regulation of postexercise transcriptional expression, with an increased requirement of HIF1α in mediating the transcriptional response to exercise at ZT3.

## Discussion

HIF1α is a key factor in the time-of-day-dependent regulation of skeletal muscle and systemic metabolism during exercise (7). Based on the increased and sustained activation of HIF1α and its target genes following exercise at ZT15, we hypothesized that the absence of skeletal muscle HIF1α would result in more pronounced disruptions to metabolic processes at this timepoint. Unexpectedly, our findings reveal that HIF1α plays a more prominent role at ZT3, as evidenced by the most substantial transcriptomic changes observed at this phase. Nevertheless, at both timepoints, HIF1α is required for the exercise-induced elevation of glycolytic metabolism, with this effect being more pronounced at ZT3. Specifically, fl/fl mice exhibited increased systemic glucose oxidation at ZT3, a response that was attenuated in HIF1α KO mice, suggesting that exercise-induced glucose metabolism is both time-of-day and HIF1α-dependent. While previous studies have reported reduced lactate production in HIF1α KO mice (24), our study further demonstrates that this metabolic shift is accompanied by an increase in mannose-6-phosphate production. Notably, this alteration occurs independently of changes in molecular or behavioral rhythmicity, presenting a robust model to explore time-of-day-dependent metabolic regulation free from confounding factors such as nutritional state or disruptions to the circadian clock.

Skeletal muscle-specific HIF1α KO mice exhibit reduced blood lactate levels following exercise and enhanced endurance capacity under basal conditions (24, 25). While no performance advantages were observed in the HIF1α KO mice after a time-of-day graded exercise test, an overall increase in voluntary wheel running was observed. Furthermore, distinct differences in metabolic responses to exercise were identified. Notably, following a 60-min steady-state bout of exercise at ZT3, fl/fl mice exhibited sustained reductions in blood glucose levels and systemic glucose oxidation, effects that were absent in HIF1α KO mice. Metabolomic profiling revealed that HIF1α regulates glucose partitioning within skeletal muscle. In fl/fl mice, glucose utilization is directed toward lactate production, whereas in HIF1α KO mice, it is redirected toward mannose-6-phosphate synthesis. This metabolic reprogramming is corroborated by the increased expression of mannose phosphate isomerase and components of the pyruvate dehydrogenase complex in HIF1α KO muscle. While the decreased lactate production, resulting from relieved inhibition of pyruvate dehydrogenase, is consistent with prior findings (26), the increased production of mannose-6-phosphate is not. Most notably known for its role in the lysosomal pathway (27), mannose-6-phosphate has also been shown to facilitate recovery in tendons and nerves by inhibiting the cytokine transforming growth factor-beta (28, 29). This may explain the increased voluntary wheel running activity in HIF1α KO compared to fl/fl mice.

The metabolic alterations observed in the HIF1α KO mice are further substantiated by distinct changes observed at the transcriptomic level following acute exercise. Notably, at ZT3, HIF1α KO mice exhibit substantial enrichment in gene sets associated with oxidative metabolism and mitochondrial function. These changes are less pronounced at ZT15, likely due to time-of-day variations in metabolic processes. For example, at ZT3, the mouse enters the rest phase in a fed state, with glycogen stores replenished, and glucose oxidation is increased (30). In contrast, at ZT15, the mouse transitions into the active phase after a period of fasting and glycogen depletion where it has been more reliant on fatty acids to support metabolism.

While the metabolic and transcriptomic findings suggest a specific role for HIF1α in mediating the glycolytic response to exercise at ZT3, evidence also supports its importance at ZT15. In oGTTs conducted following an acute exercise bout, we observed that at ZT15, the insulin-sensitizing effects of exercise were diminished in HIF1α KO mice. Specifically, exercised fl/fl mice exhibited the expected decrease in insulin excursion, with a glucose response similar to that of the sedentary controls: In contrast, HIF1α KO mice displayed an exaggerated insulin excursion, resembling the response observed in individuals on a high fat or ketogenic diet (31, 32), who also exhibit increased insulin secretion following an oral glucose bolus. A potential explanation for this phenomenon in exercised HIF1α KO mice is that the metabolic stress of exercise drives them further into an oxidative state, a shift that is prevented in fl/fl mice due to the ability to upregulate glycolytic genes via *Hif1α*.

Our results further demonstrate that the metabolic state of the organism, rather than nutritional status alone, influences the exercise response. At ZT3, when glycogen stores are replete, fl/fl mice exhibit robust glycolytic metabolism. In contrast, at ZT15, following an overnight fast, both fl/fl and HIF1α KO mice display an oxidative metabolic profile with comparable exercise responses, independent of HIF1α expression. The inability of HIF1α KO mice to fully support the glycolytic response at ZT3, despite similar glycogen levels and food intake patterns as fl/fl mice, underscores the essential role of HIF1α in linking metabolic state to time-of-day-specific exercise adaptations.

Given the established interactions between HIF1α and core circadian clock components such as BMAL1 and CRY (20, 23), we also assessed whether the absence of HIF1α in skeletal muscle perturbs diurnal behavior and metabolism. In contrast to previous studies that focused on single time-point analyses (24, 25), we conducted a comprehensive profiling of systemic and skeletal muscle-specific metabolism across the circadian cycle. Despite the known interactions between *Hif1*α and core clock regulation (21–23), molecular rhythmicity of this machinery remained unaffected in HIF1α KO mice. Markers of circadian rhythmicity, including behavioral patterns and core clock gene expression in skeletal muscle, were comparable between fl/fl and HIF1α KO mice. This response resembles observations from muscle-specific BMAL1 knockout mice, wherein altered glucose and lipid metabolism is apparent despite minimal disruption to circadian rhythms and behavioral patterns (33, 34). Considering the more robust metabolic phenotype in muscle-specific BMAL1 knockout mice compared to HIF1α knockout mice, coupled with the documented heterodimerization between BMAL1 and HIF1α in activating E-box-containing genes, it could be hypothesized that a portion of the phenotype observed in HIF1α knockout mice results from the loss of this heterodimer-mediated transcriptional activation (23). Muscle-specific BMAL1 knockout leads to reduced HIF1α expression, whereas muscle-specific HIF1α knockout does not affect BMAL1 levels. Furthermore, no significant differences are observed in gene expression after gene activation from the transcription factor heterodimer BMAL1/CLOCK compared with activation from the BMAL1/HIF1α heterodimer (23, 35). Collectively, these findings suggest redundancy in the activation of BMAL1-regulated genes, potentially attributable to BMAL1 exerting more substantial metabolic contributions than HIF1α. The preservation of circadian rhythmicity in both the muscle specific BMAL1 and HIF1α KO mice likely reflects the dominant role of the master clock in the suprachiasmatic nucleus, along with systemic signals from other tissues such as liver and adipose tissue, in maintaining circadian synchronization. Thus, while HIF1α may play a secondary role in integrating environmental stressors with circadian rhythms, its absence does not disrupt the core clock machinery under standard physiological conditions.

In summary, skeletal muscle-specific HIF1α ablation shifts systemic metabolism toward oxidative pathways and reduces reliance on glycolytic metabolism during exercise. These time-of-day specific responses, where more pronounced effects on blood glucose levels are observed at ZT3 and more marked alterations in insulin sensitivity at ZT15, underscore the role of HIF1α in modulating diurnal metabolic responses. Our study further elucidates the mechanism by which HIF1α regulates glucose partitioning, promoting glycolytic metabolism during exercise by promoting lactate production and shunting glucose away from mannose-6-phosphate. Collectively, these findings highlight the complex interplay between metabolic state, nutritional status, and circadian regulation in shaping exercise responses, providing insights into the role of HIF1α in skeletal muscle metabolism.

## Limitations of the Study

Skeletal muscle is a highly complex and heterogeneous tissue, composed of diverse cell types that collectively contribute to its functional and metabolic roles (36). While our study provides a comprehensive transcriptional profile following skeletal muscle knockout of HIF1α, some observed effects may be influenced by contributions from nonmyofiber cell populations, which could potentially obscure cell-type-specific responses. Additionally, our analyses focused primarily on the gastrocnemius and quadriceps muscles. Although these muscles are widely studied and functionally relevant, this focus may limit insights into the role of HIF1α in other muscle groups with distinct fiber composition or metabolic profiles. Finally, although both male and female mice were included in the study, the sample size was insufficient to permit a robust evaluation of sex-specific effects, which remain an important area for further investigation.

## Methods

### Ethical Statement.

Experiments were approved by the Danish Animal Experiments Inspectorate and performed according to local committee guidelines (license numbers 2018-15-0201-01493 and 2017-15-0201-1276).

### Animals.

HIF1α^flox^ mice were obtained from The Jackson Laboratory (RRID:IMSR_JAX:007561). These mice have *loxP* sites flanking exon 2 of the *Hif1α* gene. Skeletal muscle-specific deletion of *Hif1α* was generated by crossing HIF1α^flox^ homozygote mice with mice carrying the cre-recombinase driven by the human α-skeletal actin (HSA) promoter heterozygote males (37). Experimental animals were derived from crossing homozygote HIF1α^flox^ females (HSA-Cre^−/−^ and HIF1α^fl/fl^) with homozygote HIF1α^flox^ and heterozygote HSA-cre males (HSA-Cre^+/−^ and HIF1α^fl/fl^) (Fig. 1*A*). Mice were bred on a C57BL/6JBomTac (Taconic, Denmark) background for 2 to 9 generations before experimental use. All mice were group-housed with littermates of the same sex (2 to 7 in a cage) and maintained at 22 ± 1 °C under a 12:12 h light:dark cycle. Mice had ad libitum access to a chow diet (Altromin #1310) and water. Mice were housed in cages with aspen chip bedding, nesting material, environmental enrichment objects (cardboard tunnel, wooden block), and a red Perspex shelter. Male and female mice (12 to 18 wk of age) were utilized equally for experiments. All experiments were conducted at either zeitgeber time (ZT) 3 or ZT15 whereby ZT0 refers to the start of the light period, or rest phase and ZT12 represents the start of the dark period, or active phase unless otherwise stated. Experiments conducted during the dark phase were performed under red light.

### Acute Treadmill Exercise.

For treadmill running experiments, all mice (exercise and sedentary controls) were acclimatized to the treadmill (Exer3/6, Columbus Instruments) as described (7). During an acute exercise, bout mice started running at 6 m/min, and the speed was increased every 2 min by 2 m/min until a speed of 16 m/min was reached. Mice then ran at 16 m/min until a total treadmill time of 60 min was reached. During the same period, sedentary mice were placed on a mock treadmill.

Graded exercise tests started at 10 m/min for 10 min. After which, the speed increased 4 m/min every 4 min until a maximum speed of 30 m/min was reached. Mice that lagged on the charged coil for more than 10 s were removed from the treadmill. Running distance, speed, and time were recorded. Blood glucose (Contour XT, Bayer) and lactate (Lactate Pro2, AxonLab) were measured from tail-tip blood prior to and immediately post exercise.

### Glucose Tolerance Tests.

oGTTs in the basal state were conducted on mice following a 4 h fast prior to glucose delivery at either ZT3 or ZT15 (38). To assess glucose tolerance in the postexercise period, oGTTs were conducted following acute treadmill running at ZT3 or ZT15. Following treadmill running, mice were returned to the respective home cage for a period of 1 h, in which no food access was provided. Therefore, for the post ZT3 and ZT15 exercise oGTT, the delivery of glucose was performed at ZT5 and ZT17, respectively. For both basal and postexercise oGTT, mice received a bolus of glucose diluted in water, at a concentration of 2 g/kg body weight. Glucose was measured from the tail-tip (Contour XT, Bayer) in the basal state, as well as at 15-, 30-, 60-, and 90-min post glucose administration. Whole-blood samples for insulin were collected in the basal state and at 15-, 30-, and 60-min post glucose administrations as described (38). iAUC was calculated by the trapezoidal method subtracting the baseline.

### Indirect Calorimetry.

The Sable Systems Promethion Core was utilized for metabolic chamber experiments. One cohort of mice was utilized for basal measurements and a second cohort for voluntary wheel running assessment. The first 3 d were counted as acclimatization, with data being analyzed from the proceeding 5 d. Gas exchange measurements were taken every minute and averaged over hourly intervals for analysis. Mice who ran less than 100 m per day were excluded from the voluntary running wheel analysis.

### In Vivo ^13^C Glucose Oxidation Assay.

Mice received ^13^C-labeled glucose [U-^13^C] (2 mg/mouse in 100 μL saline) administered by gavage immediately following treadmill or mock treadmill intervention at ZT3 and were placed into cages in the Sable Systems Promethion Core, which was connected to the Stable Isotope Gas Analyzer (Sable Systems International). Analysis of expired ^13^CO_2_ was measured over the subsequent 2 h, whereby glucose oxidation was calculated using area under the curve.

### In Vivo ^13^C Glucose Metabolomics Assay.

Mice underwent surgery for jugular vein catheters with 7 d recovery prior to experiment. ^13^C-labeled glucose [U-^13^C] (30 mg/mouse) was administered via vascular access button immediately prior to treadmill or mock treadmill intervention at ZT3. Twenty minutes later mice were killed, and tissues were collected. This duration was selected based on previous studies showing significant isotopologue enrichment occurring around this timepoint (39, 40). The powdered tissues were dissolved in 200 µL of an ice-cold 90% methanol (Thermo Fisher Scientific, Waltham, MA) including 10 mg/L nonanoic acid and 10 mg/L 2-deoxy-D-glucose-6-phosphate (Sigma-Aldrich, St. Louis, MO) as an internal standard. Ceramic beads were added to the tissues prior to tissue-lysing using a TissueLyser (Qiagen, Hilden, Germany). The samples were then transferred to reaction tubes (Eppendorf, Eppendorf, Germany). The suspension was incubated on ice for 1 h for protein precipitation. Then, the samples were centrifuged at 12,000 rpm for 15 min at 4 °C. The methanolic extracts were transferred to new reaction tubes and stored at −80 °C until ready for derivatization. The samples were derivatized in two ways to increase the sensitivity of the mass spectrometry measurement and improve the chromatographic separation. One part derivatized with 3-Nitrophenylhydrazine (3NPH), N-(3-dimethylaminopropyl)-N′-ethylcarbodiimide hydrochloride (EDC) and Butylhydroxytoluene (BHT) (all from Sigma-Aldrich) for carboxylic acids, and one following a method described by Rende et al for sugar phosphates (41). For the carboxylic acid derivatization, in HPLC vials with 300 µL inserts (Thermo Fisher Scientific), 20 μL of supernatant extracts were combined with 20 μL of a 3NPH solution (200 mM in 50% methanol) and 20 μL of an EDC solution (120 mM in 50% methanol and 6% pyridine) and briefly vortexed. After 1 h incubation at room temperature, 40 μL of a BHT solution (0.05 mg*mL^−1^ in methanol) were added and briefly vortexed. For the sugar phosphates, in HPLC vials with 300 µL inserts (Thermo Fisher Scientific), 50 µL of the samples were dried before being oxomylated followed by propionylation. The dried extracts were reconstituted in 20 µL MOX (Thermo Fisher Scientific) and incubated at 60 °C for 1 h. Then, 6 µL 1-Methylimidazole (Sigma Aldrich) were added as a catalyst, together with 12 µL of the reactant, propionic anhydride (Sigma Aldrich), before incubating for 30 min at 37 °C. Samples were dried for 30 min and reconstituted in water with 0.1% formic acid. The samples were kept at 4 °C until analysis on the same day.

The LC/MS-qTOF analysis for the carboxylic acids was performed using an Agilent 1290 II Liquid chromatograph (Agilent, Santa Clara, CA) with an Acquity HSS T3 10 cm × 2.1 mm × 1.8 μm column (Waters, Milford, MA) with an Acquity HSS T3 5 mm × 2.1 mm × 1.8 µm VanGuard precolumn. Then, an Impact ii quadrupole time-of-flight mass spectrometer (Bruker, Bremen, Germany) equipped with a VIP-ion source was used for detection and TASQ 2023b (Bruker) for data extraction. Water (Honeywell, Charlotte, NC) with added 0.1% formic acid (Thermo Fisher Scientific) was used for mobile phase A, acetonitrile (Honeywell) with 0.1% formic acid was used for mobile phase B. The mobile phase gradient started with 5% of mobile phase B and increased to 100% of mobile phase B over the course of 12 min and remained at 100% for 1 min before being decreased again to initial conditions over the course of 30 s where it remained for 2 min 30 s. The column oven temperature was kept at 40 °C, the multisampler cooled to 6 °C. For the sugar phosphates, the same type of chromatograph, source, and data processing software was used with an Acquity Premier BEH C18 VanGuard FIT 10 cm × 2.1 mm × 1.7 µm column (Waters), measured on a TimsTOF Pro 2 (Bruker), with the following separation mode. Solvent A: water with 0.1% formic acid. Solvent B: methanol with 0.1% formic acid. Data were acquired from 0 min to 17 min 30 s. The following gradient was applied: 0 min 0% B, from 0 min to 1 min 0% B, from 1 min to 3 min increase to 30% B, from 3 min to 6 min increase to 40% B, from 6 min to 10 min 40% B, from 10 min to 12 min 30 s increase to 70% B, from 12 min 30 s to 15 min 70% B, from 15 min to 17 min increase to 99% B, from 17 min to 18 min 99%, from 18 min to 18 min 30 s to 0% B, maintained until 20 min, with a flow of 0.5 mL/min. The column oven temperature was kept at 55 °C, the multisampler cooled to 6 °C. For both measurements, the injection volume was 1 μL. The acquisition was performed in full scan negative mode in the mass range from 50 to 1,000 m/z at 2 Hz. Sodium formate was used as an internal calibrant. The TASQ method for data extraction was previously set up based on retention times and m/z values of derivatized standards. The data were corrected for natural abundance of carbon 13 using IsoCor and processed and analyzed using custom Python scripts (42). Isotopologue values underwent Tukey’s Ladder transformation when data were nonnormally distributed. Two-way ANOVAs were performed using “lm” to test for the main effects of genotype and treatment, and for a genotype x treatment interaction in each tissue and metabolite. Where significant, post hoc testing was conducted using “emmeans” (43).

### Tissue Collection.

For basal conditions, tissue collections were performed at ZT 0, 4, 8, 12, 16, and 20 whereby ZT 0 refers to the onset of the light cycle. For exercise-stimulated conditions, tissue collections were performed immediately post (0 h) and 3 h post–acute treadmill exercise. Mice were anesthetized (confirmed by lack of pedal-reflex) via intraperitoneal injection of pentobarbital (100 mg/kg body weight) and exsanguinated via cardiac puncture immediately followed by cervical dislocation. Plasma was collected from whole blood that was subjected to centrifugation at 2,000 g for 10 min at 4 °C. Muscle and liver tissue were immediately snap frozen after dissection into liquid nitrogen and subsequently stored at −80 °C.

### Plasma Assays.

Insulin was analyzed with an ELISA (Ultra-Sensitive Mouse Insulin ELISA kit, Crystal Chem, #90080). Triglycerides were measured with a colorimetric assay (Randox). NEFA was determined with a NEFA HR(2) kit (Wako).

### Tissue Glycogen Quantification.

The amyloglucosidase method was utilized to measure tissue glycogen content. Approximately 30 mg of powdered tissue was digested in 1 M KOH (200 µL) at 70 °C for 20 min. Saturated Na_2_SO_4_ (75 µL) and absolute ethanol (1.7 mL) were added to the tissue prior subjecting the sample centrifugation at 18,000 g for 15 min at 4 °C. The pellet was resuspended in distilled water (200 µL) and incubated for 10 min at 70 °C. Absolute ethanol was added (1.8 mL) followed by subjecting the sample to centrifugation as described above. The pellet was resuspended in acetic acid (1 mL; 0.25 M, pH 4.75) with amyloglucosidase (1 unit per mL of buffer; Sigma A1602). Samples were incubated overnight at 37 °C with shaking (800 rpm). Glucose was measured with the colorimetric assay (GOD-PAP; Randox).

### RNA Extraction.

Muscle tissue (gastrocnemius; ~30 mg) was homogenized in Trizol (1 mL) using a steel bead with the Qiagen TissueLyser II (3 × 90 s at 30 Hz; 4 °C). Chloroform (200 µL) was added to all the samples and mixed by 30 s of shaking prior to centrifugation for 15 min at 12,000 g at 4 °C. The aqueous phase was combined with an equivalent volume of 70% ethanol and thereafter RNA extraction was performed according to the manufacturers’ instructions for the RNeasy Mini Kit (74,106, Qiagen). RNA quantification was done with a spectrophotometer (Nanodrop 8000, Thermo Scientific).

### cDNA Synthesis, and qPCR.

Copy DNA (cDNA) synthesis was performed using 1 μg of RNA (Bio-Rad iScript cDNA synthesis kit). qRT-PCR was performed using Sybr Green (Brilliant III) on a Bio-Rad CFX384 Real-Time PCR system. Primers (found in *SI Appendix*, Table S1) were used at a final concentration of 200 nM. Relative quantification was determined using the ddCCT method with the housekeeping gene *18S* (44).

### Library Preparation and RNA-Sequencing.

RNA (gastrocnemius muscle) quality was assessed using a Bioanalyzer instrument (Agilent). Libraries were prepared using 500 ng of RNA and the TruSeq Stranded mRNA Library Prep assay kit. Poly-A containing mRNA molecules were purified using poly T-oligo-attached magnetic beads, fragmented and cDNA synthesized using SuperScript III Reverse Transcriptase (Thermo Fisher Scientific). Synthesized cDNA was adenylated, ligated to single-end adapters and underwent a clean-up using AMPure XP beads (Beckman Coulter). DNA fragments were amplified using PCR followed by a final clean-up. Libraries were subjected to 52-bp paired-end sequencing on the Nova Seq 6000 instrument (Illumina).

### Bioinformatic Analysis.

Reads were aligned to the mouse genome assembly (mm10) using STAR aligner (45), and transcripts counted using featureCounts (46). One sample was excluded due to low coverage (group: HIF1α-mKO, exercise, ZT 3). Sequencing depth ranged from 24 to 40 million reads, with a mean of 16 million reads. Lowly expressed genes were removed through independent filtering using the mean of normalized counts as a filter statistic in “DESeq2,” with 20,224 genes passing the independent filtering threshold. Differential gene expression analysis was performed using “DESeq2,” (47) negative binomial generalized linear modeling and a design of *~0 + group + sex*. Gene ontology (GO) enrichment analysis was conducted using “clusterprofiler” and the biological function GO term, with all detected genes serving as background (48). All *P*-values were corrected for multiple comparisons using false discovery rate (FDR) and the alpha value was set to 0.1. Transcriptomic data are deposited under the accession number GSE282641. All code used for analysis and figure generation is located at https://github.com/kirstin-macgregor/HIF1a_timeOfDay_exercise.

### Statistical Analysis.

Data are presented as bars representing the mean with error bars representing SE. Individual data points represent individual animals. A *P*-value of less than 0.05 was considered statistically significant. Two-way ANOVAs were performed to test for a main effect of genotype and time, and for interaction effect between genotype and time. Three-way ANOVAs were performed to test for main effects of genotype, exercise, and time, as well as interactions between factors. The Sidak’s post hoc test was performed if there were interactions. ANOVAs were performed in GraphPad Prism (Version 10.1.11). Rhythmicity analysis was performed using RAIN (49) package, with independent method, a period of 24 h and time interval between samples of 4 h. Differential rhythmicity between conditions was conducted on rhythmic genes using DODR (50) with a period of 24 h. *P* values were adjusted for multiple comparisons using the Benjamini–Hochberg method and an adjusted *P* value below 0.05 was considered rhythmic.

## Supplementary Material

Appendix 01 (PDF)

## Data Availability

Transcriptomics data and code have been deposited in the Gene Expression Omnibus (GEO) under accession number GSE282641 (51). The code used for analysis and figure generation is available at https://github.com/kirstin-macgregor/HIF1a_timeOfDay_exercise (52). Anonymized transcriptomics data have also been deposited in GSE282641 (51). All other data are included in the manuscript and/or *SI Appendix*.
