# Supplementary material for: HIF1α mediates circadian regulation of skeletal muscle metabolism and substrate preference in response to time-of-day exercise
Source: Proc Natl Acad Sci U S A. 2025 Jul 8;122(28):e2504080122. doi: 10.1073/pnas.2504080122 (PMC12280960; doi:10.1073/pnas.2504080122)
Supplement: Supplementary file 1 — Appendix 01 (PDF) [file pnas.2504080122.sapp.pdf]

PNAS

Supporting Information for

HIF1α Mediates Circadian Regulation of Skeletal Muscle Metabolism and Substrate Preference in Response to Time-of-Day Exercise

Amy M. Ehrlich, Kirstin A. MacGregor, Stephen Ashcroft, Lewin Small, Ali Altıntaş, Alexander V. Chibalin, Matthias Anagho-Mattanovich, Ben Stocks, Thomas Moritz, Jonas T. Treebak, Juleen R. Zierath

Juleen R. Zierath
Email: juleen.zierath@ki.se

This PDF file includes:

Figures S1 to S7
Tables S1

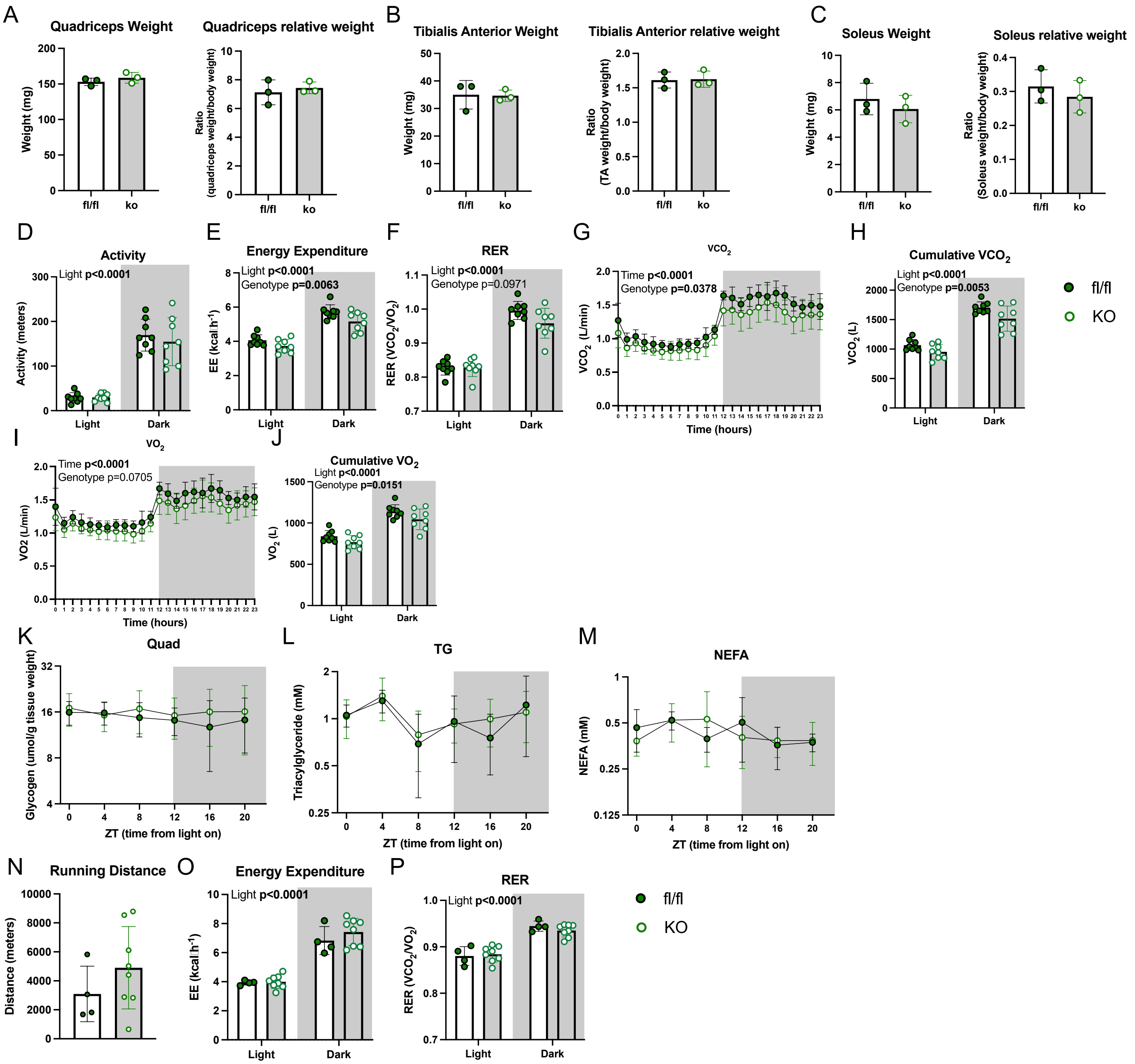
A
Quadriceps Weight
Weight (mg)
Quadriceps relative weight
Ratio (quadriceps weight/body weight)
B
Tibialis Anterior Weight
Tibialis Anterior relative weight
Ratio (TA weight/body weight)
C
Soleus Weight
Soleus relative weight
Ratio (Soleus weight/body weight)
fl/fl
ko
D
Activity
Light p<0.0001
Activity (meters)
E
Energy Expenditure
Light p<0.0001
Genotype p=0.0063
EE (kcal h-1)
F
RER
Light p<0.0001
Genotype p=0.0971
RER (VCO2/VO2)
G
VCO2
Time p<0.0001
Genotype p=0.0378
VCO2 (L/min)
Time (hours)
H
Cumulative VCO2
Light p<0.0001
Genotype p=0.0053
VCO2 (L)
Light
Dark
fl/fl
KO
I
VO2
Time p<0.0001
Genotype p=0.0705
VO2 (L/min)
Time (hours)
J
Cumulative VO2
Light p<0.0001
Genotype p=0.0151
VO2 (L)
K
Quad
Glycogen (umol/g tissue weight)
ZT (time from light on)
L
TG
Triacylglyceride (mM)
ZT (time from light on)
M
NEFA
NEFA (mM)
ZT (time from light on)
N
Running Distance
Distance (meters)
O
Energy Expenditure
Light p<0.0001
EE (kcal h-1)
P
RER
Light p<0.0001
RER (VCO2/VO2)
fl/fl
KO

Fig. S1.

A) Quadriceps absolute weight (mg) and quadriceps relative weight calculated by taking the ratio of quadriceps weight (mg) and body weight (g).
B) Tibialis anterior absolute weight (mg) and tibialis anterior relative weight calculated by taking the ratio of tibialis anterior weight (mg) and body weight (g).
C) Soleus absolute weight (mg) and soleus relative weight calculated by taking the ratio of soleus weight (mg) and body weight (g).
D) Average sum of activity occurring in the light phase and the dark phase.
E) Average sum of energy expenditure occurring in the light phase and in the dark phase.
F) Average hourly RER in the light phase and in the dark phase.
G) Trace of average hourly carbon dioxide production per hour over a 24-hour period.
H) Sum of carbon dioxide production during the light phase and in the dark phase.
I) Trace of average oxygen consumption per hour over a 24-hour period.
J) Sum of oxygen consumption during the light phase and in the dark phase.
K) Glycogen content in quadriceps muscle measured every 4 hours over a 24-hour period.
L) Plasma triglycerides measured every 4 hours over a 24-hour period.
M) Plasma non-esterified fatty acids measured every 4 hours over a 24-hour period.
N) Average daily running distance on voluntary running wheels.
O) Average sum of energy expenditure in the light and dark phase when mice have access to voluntary running wheels.
P) Average RER in the light and dark phase when mice have access to voluntary running wheels.

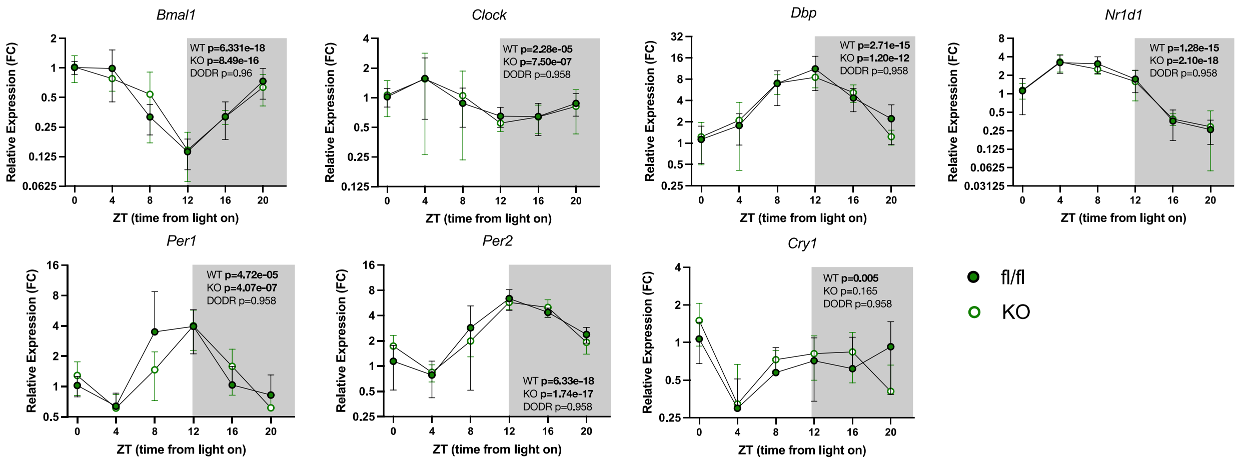

Fig. S2. Clock gene expression in quadriceps muscle measured every 4 hours over a 24-hour period.

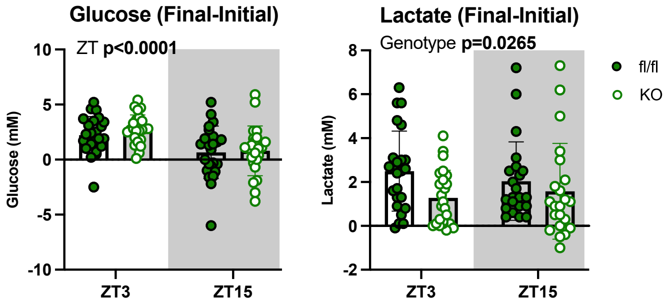

Fig. S3. Change in blood glucose and lactate levels from the pre to post exercise test.

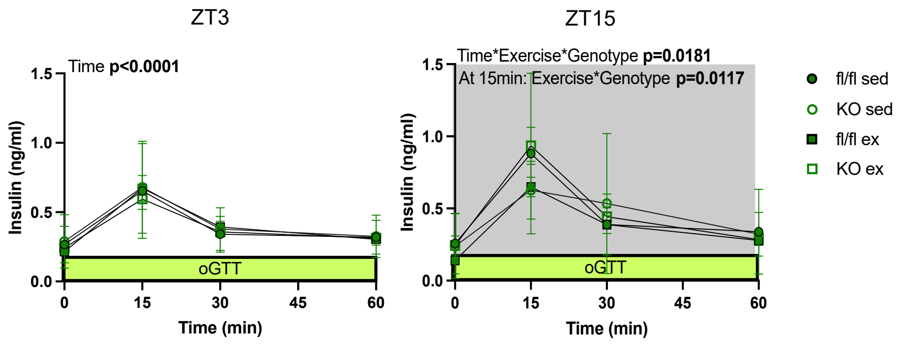

Fig. S4. Blood insulin measurements during an oral glucose tolerance test at ZT3 and ZT15 following an acute exercise bout.

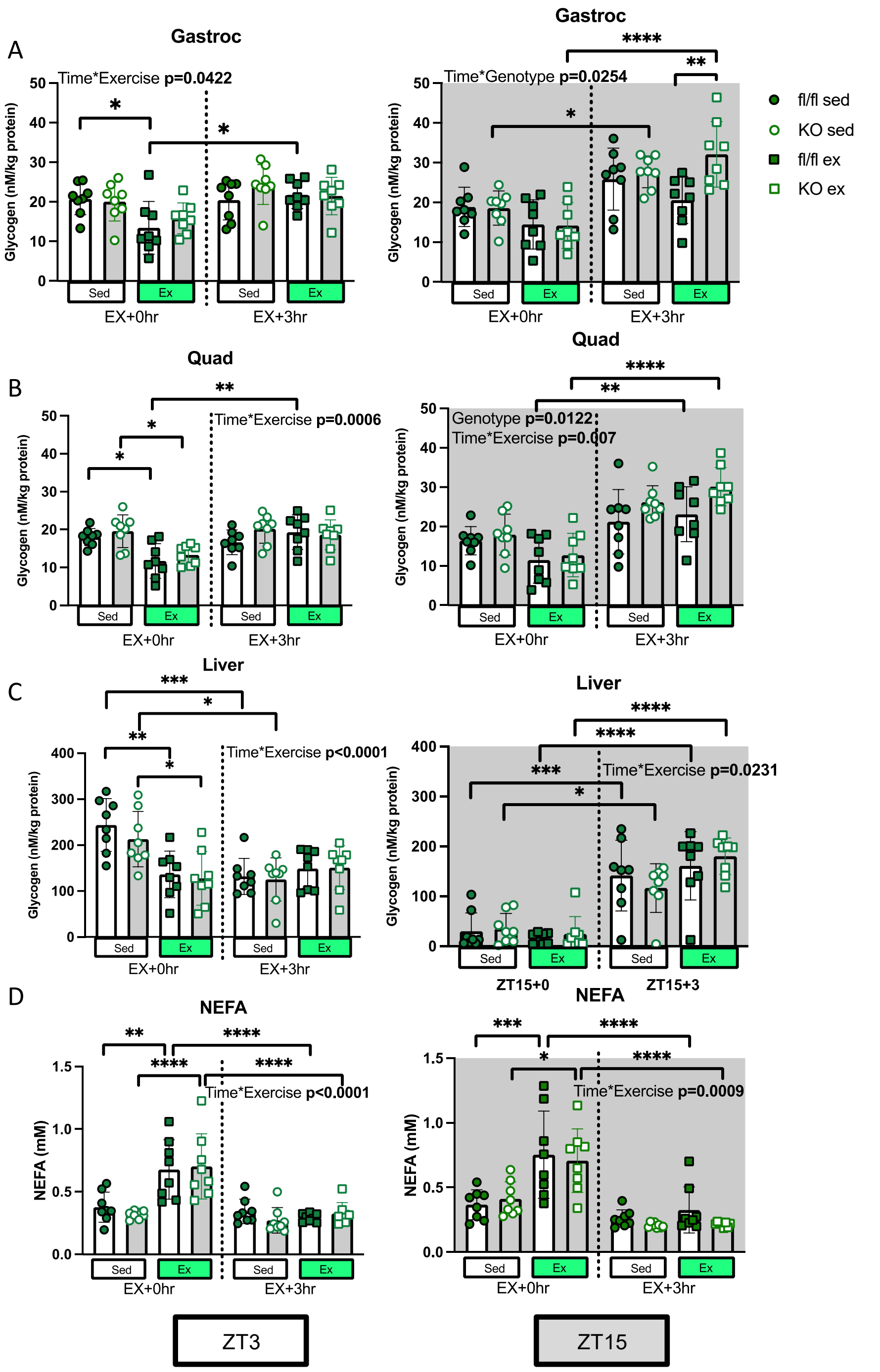
A
B
C
D
Gastroc
Quad
Liver
NEFA
Time*Exercise p=0.0422
Time*Genotype p=0.0254
Time*Exercise p=0.0006
Genotype p=0.0122
Time*Exercise p=0.007
Time*Exercise p<0.0001
Time*Exercise p=0.0231
Time*Exercise p<0.0001
Time*Exercise p=0.0009
Glycogen (nM/kg protein)
NEFA (mM)
Sed
Ex
EX+0hr
EX+3hr
ZT15+0
ZT15+3
fl/fl sed
KO sed
fl/fl ex
KO ex
ZT3
ZT15

Fig. S5.

A) Gastrocnemius muscle glycogen content at ZT3 and ZT15 measured immediately after an acute exercise bout (60 minutes) and measured 3 hours after the exercise bout.
B) Quadriceps muscle glycogen content at ZT3 and ZT15 measured immediately after an acute exercise bout (60 minutes) and measured 3 hours after the exercise bout.
C) Liver glycogen content at ZT3 and ZT15 measured immediately after an acute exercise bout (60 minutes) and measured 3 hours after the exercise bout.
D) Plasma NEFA concentration at ZT3 and ZT15 measured immediately after an acute exercise bout (60 minutes) and measured 3 hours after the exercise bout.

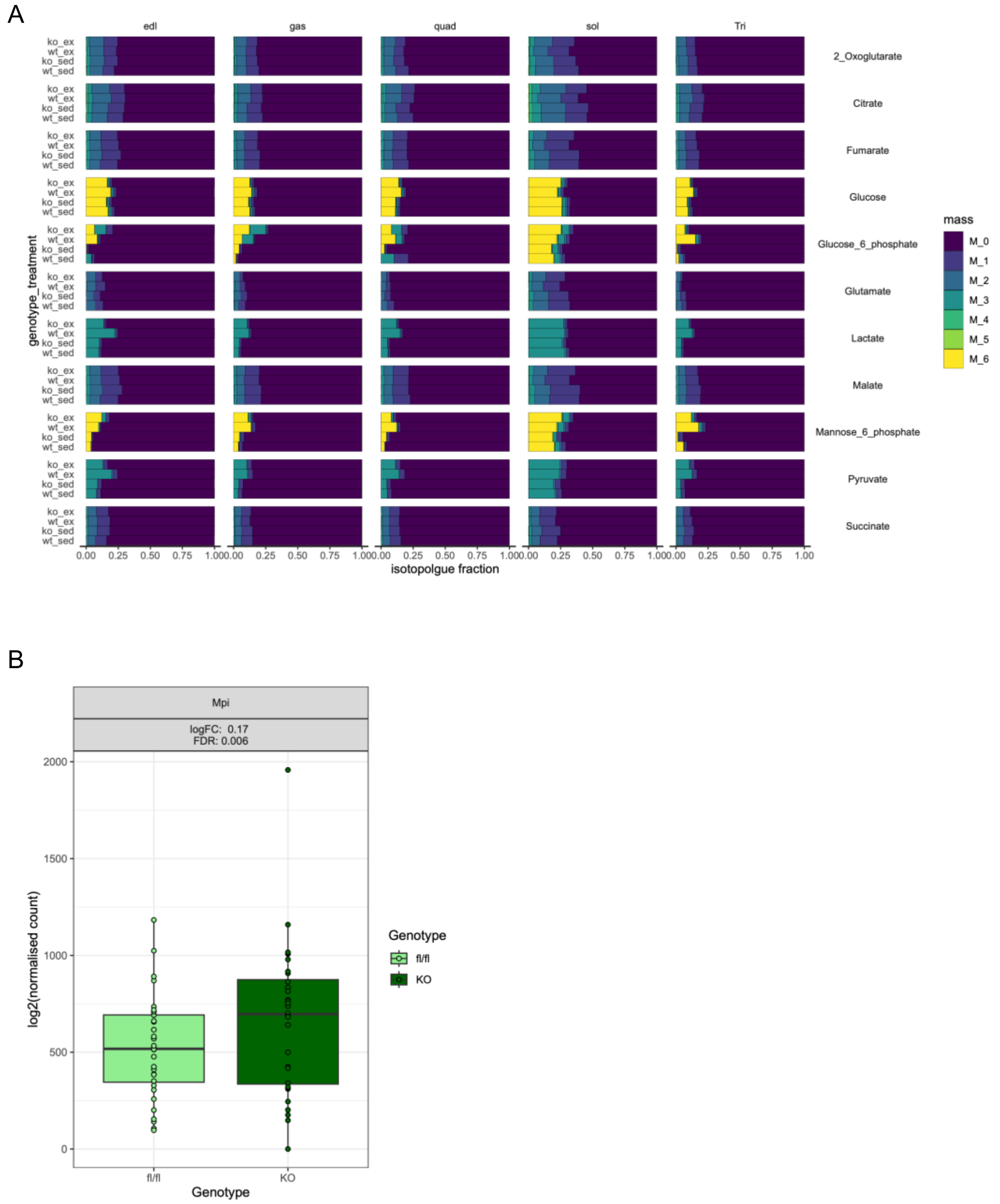

Fig. S6.

A) Summary of the changes in 13C fractions of metabolites in Extensor Digitorum Longus (EDL). Gastrocnemius, quadriceps, soleus and triceps muscles following 13C-glucose administration in HIf1a KO and WT mice.

B) Mpi gene expression in the gastrocnemius

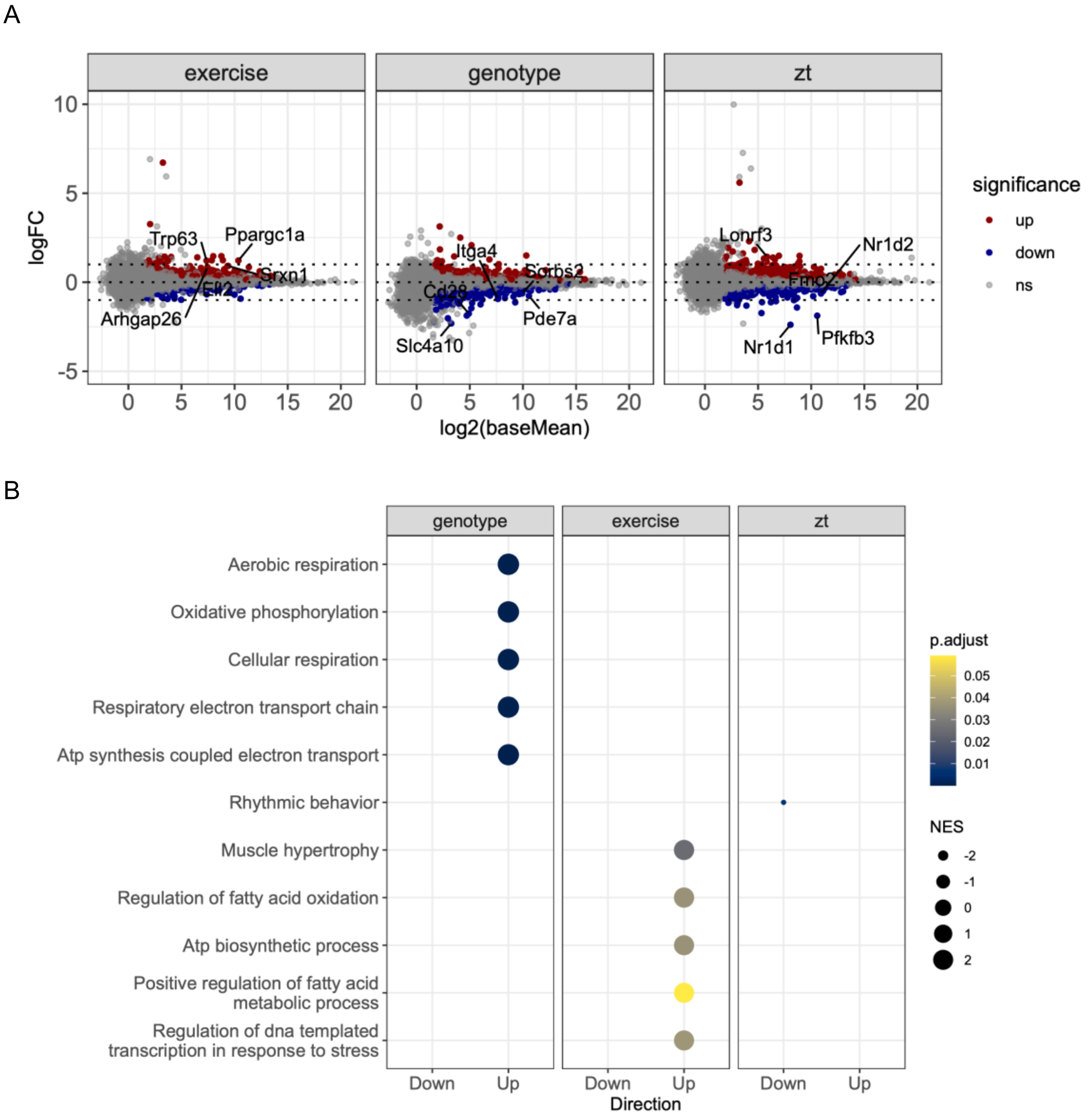

Fig. S7.

A) MA plot showing the log average expression against the log fold change for the main effect of genotype, exercise and ZT. The top 10 differentially expressed genes are labelled.

B) Gene ontology biological processes for the main effect of genotype, exercise and zt. Gene set enrichment analysis was performed on genes ranked by FDR.

Gene	Forward (5’ to 3’)	Reverse (5’ to 3’)
**18S**	AGT CCC TGC CCT TTG TAC ACA	GAT CCG AGG GCC TCA CTA AAC
**Hif1α**	AGCTTCTGTTATGAGGCTCACC	GTCCATCTGTGCCTTCATCTCA
**Bmal1**	TAGGATGTGACCGAGGGAAG	TCAAACAAGCTCTGGCCAAT
**Clock**	CGTCCTTCAGCAGTCAGTCCA	TTGCAGCTTGAGACATCGCT
**Nr1d1**	GTCTCTCCGTTGGCATGTCT	CCAAGTTCATGGCGCTCT
**DBP**	AATGACCTTTGAACCTGATCCCGCT	GCTCCAGTACTTCTCATCCTTCTGT
**Per1**	TCAGAGTCCCAGACCAGGTGTC	GGTGCACAACGGGGCTTTTG
**Per2**	AATGGCCAAGAGGAGTCTCA	ATGCTTCCTTCTGTCCTCCA
**Cry1**	AGCGCAGGTGTCGGTTATGAGC	ATAGACGCAGCGGATGGTGTCG

Table S1. Primers used in this study
